# Intraspecific divergence within *Microcystis aeruginosa* mediates the dynamics of freshwater harmful algal blooms under climate warming scenarios

**DOI:** 10.1098/rspb.2024.2520

**Published:** 2025-02-05

**Authors:** Mirte C. M. Kuijpers, Catherine V. Quigley, Nicole C. Bray, Wenbo Ding, Jeffrey D. White, Sara L. Jackrel

**Affiliations:** ^1^ Department of Ecology, Behavior and Evolution, School of Biological Sciences, University of California San Diego, La Jolla, CA, USA; ^2^ Department of Biology, Framingham State University, Framingham, MA, USA

**Keywords:** intraspecific diversity, climate warming, cyanobacterial harmful algal blooms, local adaptation, heat shock protein gene expression

## Abstract

Intraspecific biodiversity can have ecosystem-level consequences and may affect the accuracy of ecological forecasting. For example, rare genetic variants may have traits that prove beneficial under future environmental conditions. The cyanobacterium responsible for most freshwater harmful algal blooms worldwide, *Microcystis aeruginosa*, occurs in at least three types. While the dominant type occurs in eutrophic environments and is adapted to thrive in nutrient-rich conditions, two additional types have recently been discovered that inhabit oligotrophic and eutrophic environments and have genomic adaptations for survival under nutrient limitation. Here, we show that these oligotrophic types are widespread throughout the Eastern USA. By pairing an experimental warming study with gene expression analyses, we found that the eutrophic type may be most susceptible to climate warming. In comparison, oligotrophic types maintained their growth better and persisted longer under warming. As a mechanistic explanation for these patterns, we found that oligotrophic types responded to warming by widespread elevated expression of heat shock protein genes. Reduction of nutrient loading has been a historically effective mitigation strategy for controlling harmful algal blooms. Our results suggest that climate warming may benefit oligotrophic types of *M. aeruginosa*, potentially reducing the effectiveness of such mitigation efforts. In-depth study of intraspecific variation may therefore improve forecasting for understanding future whole ecosystem dynamics.

## 1. Introduction

Variation within a species can rival the effects of variation among species in regulating whole ecosystem dynamics, including biogeochemical cycling [1,2]. Standing genetic variation within a species can also be a critical resource when leveraged in response to changing environmental conditions. Using a species that is known to have widespread ecosystem level effects under current environmental conditions, we investigate how standing intraspecific variation may elucidate future ecosystem-level impacts of a species under climate warming.

Cyanobacteria (formerly blue-green algae) are a clade of photosynthetic prokaryotes and the oldest known oxygen-producing organisms on Earth, with fossils dating back to before the great oxidation event [3]. The clade is found across a diversity of environments, including eutrophic and oligotrophic lakes and tropical and polar regions [4,5]. Many species can form dense, and sometimes toxic, blooms which visibly discolour their freshwater, brackish or marine environment and often have negative impacts on their ecosystem [6]. These blooms also incur significant costs to tourism, agriculture, and human health [7].

The cyanobacterial clade has survived major changes in the Earth’s climate and bloom-forming species now seem to be benefiting from the effects of anthropogenic climate change [6,8]. This includes more extreme precipitation (which can lead to increased nutrient run-off into aquatic systems), droughts (which can lead to increased retention time of cyanobacteria within lakes) and, critically, increased temperatures (which often give cyanobacterial species a competitive advantage over eukaryotic algae) [8]. The advantages of higher temperatures to bloom-forming cyanobacteria are twofold. First, higher temperatures lead to increased stratification of warmer water which favours buoyant cyanobacterial species while causing increased sedimentation of non-buoyant eukaryotic algal species [9]. Second, many cyanobacteria have a competitive growth advantage at higher temperatures, with optimal growth rates for cyanobacterial species often above 25°C [10,11]. Cyanobacterial blooms may even create a positive feedback loop in which warmer waters promote blooms, which, due to highly concentrated photo-absorption within the dense cyanobacterial blooms, leads to further warming of the surface waters [12,13]. A better understanding of the interaction of harmful bloom-forming cyanobacteria and climate warming will therefore be important for the prediction and mitigation of such harmful blooms in the future.

*Microcystis aeruginosa* is a widely distributed cyanobacterium that produces the hepatotoxin microcystin [14]. Toxic blooms of *M. aeruginosa* have caused mass wildlife mortality events and have threatened human drinking water supplies [14–17]. *Microcystis aeruginosa* was previously found to exist as at least three distinct genotype–environment groups (hereafter 'bacterial types') across lakes spanning a wide range of phosphorus levels in MI, USA [5]. The first bacterial type, which we refer to as high-nutrient lake/high-nutrient genotype or HL/HG, encompasses strains found in nutrient-rich conditions and contains the most well-known type of *M. aeruginosa* identified in eutrophic systems throughout the world. There is extensive genotypic diversity within this type, which has been well described previously [5]. A second bacterial type, which we refer to as LL/LG (low-nutrient lake/low-nutrient genotype), is restricted to oligotrophic, nutrient-poor ecosystems while the third bacterial type, which we refer to as HL/LG (high-nutrient lake/low-nutrient genotype), is most phylogenetically related to LL/LG but in fact occurs in eutrophic and mesotrophic lakes. The HG versus LG classifications were originally determined using metagenome similarity-based clustering, with the genomes in the LG cluster showing signs of genome streamlining [5]. The HL classification is assigned to strains isolated from eutrophic and mesotrophic lakes, whereas the LL classification is assigned to strains isolated from oligotrophic lakes using a 10 µg l^−1^ total phosphorus (TP) threshold for the oligotrophic–mesotrophic boundary [18]. Overall, this classification of *M. aeruginosa* strains into these three types is well supported by (i) a highly resolved multi-gene phylogeny, (ii) clustering of genome characteristics (particularly enrichment of genome streamlining traits in the oligotrophic types) and (iii) strong similarity within types in functional capability, as determined by a genome-wide protein functional analysis using shotgun metagenomics [5]. Our prior work suggests that the HL/LG bacterial type takes advantage of low-nutrient microenvironments within eutrophic and mesotrophic lakes, as both LL/LG and HL/LG strains show adaptations that would facilitate survival in low-nutrient conditions [5].

Harmful blooms of *M. aeruginosa* are thought to be primarily driven by excess nutrient loading, with reduction in phosphorus run-off into freshwater systems often proving successful in bloom reduction [19]. Populations of *M. aeruginosa* can experience stress from both N and P limitation, particularly during established late-stage phases of blooms [16]. Therefore, oligotrophic types may be situated to thrive within the later stages of blooms. Concerningly, *M. aeruginosa* appears not only to have a competitive growth advantage but also to produce more toxins at higher temperatures [8]. Harmful blooms of *M. aeruginosa* may therefore become more frequent and severe with climate change, and indeed, this may already be occurring in one of the largest lakes in China, Lake Taihu [17].

While *M. aeruginosa* has a growth advantage at higher temperatures, increased temperatures are a cellular stressor that will become detrimental to *M. aeruginosa* above a certain threshold. Specifically, increased temperatures can promote the misfolding of newly synthesized proteins, as well as denaturation and damage to existing proteins [20]. The heat shock proteins (HSPs) are a highly conserved and ubiquitous family of molecular chaperones, which can increase stress tolerance by preventing protein misfolding and preserving protein homeostasis under stress [21]. While constitutive HSPs are expressed at low levels under normal conditions, the expression of inducible forms of HSPs is greatly increased at elevated temperatures [22]. Thermotolerance is characterized by elevated expression of inducible forms of HSPs [23]. Not only does *M. aeruginosa* have higher optimal temperatures than many eukaryotic algae, but the cyanobacterium may also show increased tolerance to higher temperatures due to frequent exposure to elevated temperatures caused by photon-absorption of dense blooms near the lake surface [13]. We, therefore, hypothesize that certain types of *M. aeruginosa*, such as those occurring in late-stage blooms, might show elevated expression of HSPs as a means for withstanding elevated temperatures.

Understanding the effects of warming on different types of *M. aeruginosa* will be essential to predict the frequency, duration and intensity of harmful blooms under climate warming. Here, we isolated strains of *M. aeruginosa* from lakes of varying trophic status across the midwestern and northeastern USA. We found that oligotrophic types previously identified in a small region of Michigan are widespread throughout a much broader geographic region, expanding the relevance of these oligotrophic types for harmful algal blooms [5]. We then employed strains from each of the three types of *M. aeruginosa* in an experimental warming study, using four temperatures ranging from 20°C to 32°C, with the aim of evaluating the growth responses of strains belonging to each type under elevated temperatures. Next, we investigated the mechanisms of thermotolerance within each type by analysing the expression of HSPs during the warming study. Additionally, we evaluated the expression of one of the genes in the microcystin (*mcy)* operon, which is responsible for production of the hepatotoxin microcystin. Overall, our results clarify the current and potentially future relevance of oligotrophic types of *M. aeruginosa*, contributing to our ability to predict and manage harmful algal blooms under climate warming.

## 2. Methods

### (a) Isolation and culturing of *M. aeruginosa*

Sampling and isolation methods are fully described elsewhere [5,24]. In brief, we collected *M. aeruginosa* and water samples for nutrient analysis (electronic supplementary material, table S1) from lakes spanning a large productivity gradient (5.8−65 µg l^−1^ TP) in the midwestern and northeastern USA from July to August 2019. *Microcystis* colonies were isolated from water samples via sequential pipette transfers using sterile 0.5× WC-S medium and a dissecting microscope [25]. These ‘washed’ colonies were inoculated into 20 ml volumes of media. Only colonies with a distinctive shape and compact cell arrangement were selected rather than loose aggregations of cells. Successfully established isolates were maintained at 21°C in 200 ml cultures under a 12:12 h light:dark cycle with 80 μmol m^−2^ s^−1^ fluorescent lighting. Inoculums of each culture were transferred monthly to fresh media. While these cultures are not axenic, only closely associated bacteria should have remained after the initial isolation procedure [26]. See methodology for strain genotyping and phylogenetics analyses in the electronic supplementary material.

### (b) Warming experiment

We acclimated one biological replicate of each of 20 of our 24 isolated *M. aeruginosa* strains (electronic supplementary material, table S1) to four temperatures, 20°C, 24°C, 28°C and 32°C, by gradually adjusting the temperature over 48 h. We chose a single biological replicate per strain to focus our experimental design on bacterial types. We consider each strain as an independent replicate of the same bacterial type, supported by previous work showing strong genomic similarities between strains of the same type. Four deionized water baths were set up within a refrigerated incubator (Percival) with an ambient internal chamber of 20°C. Heated treatments were warmed to their desired targets and maintained by submersible heaters. Each acclimated strain × temperature combination was inoculated into 125 ml flasks of 0.5× WC-S medium and immersed in their respective bath. To minimize variation in initial cell densities across treatments, we targeted an *M. aeruginosa* biomass equivalent to 1 µg l^−1^ chlorophyll-*a*, which is a routinely used surrogate for phytoplankton biomass. Cultures were grown without media replacement under a 12:12 h light:dark cycle throughout the experiment. Biomass was subsampled weekly for the duration of the 35 day experiment by vacuum-filtering 10 ml aliquots per flask onto 23 mm A/E glass fibre filters (Pall). Filters were immediately frozen until used to determine *M. aeruginosa* growth via fluorometric analysis of chlorophyll-*a* following a 24 h dark extraction in cold 90% ethanol, with acidification (Turner Designs) [24]. On day 35, a second replicate filter was collected and stored long term at −80°C until RNA extraction.

### (c) Quantifying gene expression

We extracted RNA with Invitrogen *mir*Vana miRNA Isolation Kits following the protocol modifications of Fortunato & Huber [27]. We synthesized cDNA using the SuperScript III First-Strand Synthesis System and random hexamer primers. Quantitative real-time PCR was then performed for *M. aeruginosa* gene targets that are involved in heat stress responses (ClpB, DnaJ, DnaK1, DnaK3, DnaK-fp, GroEL, GroES, GrpE, HrcA, Hsp20, HspA, HtpG) and the reference housekeeping gene *rpoA*, using custom primers designed for *M. aeruginosa* [28]; as well as the gene *mcyE* that correlates with toxin production using HEP primers [29–31]. See methods in electronic supplementary material for details of gene expression analysis and all statistical analyses.

## 3. Results

**Figure 1.**
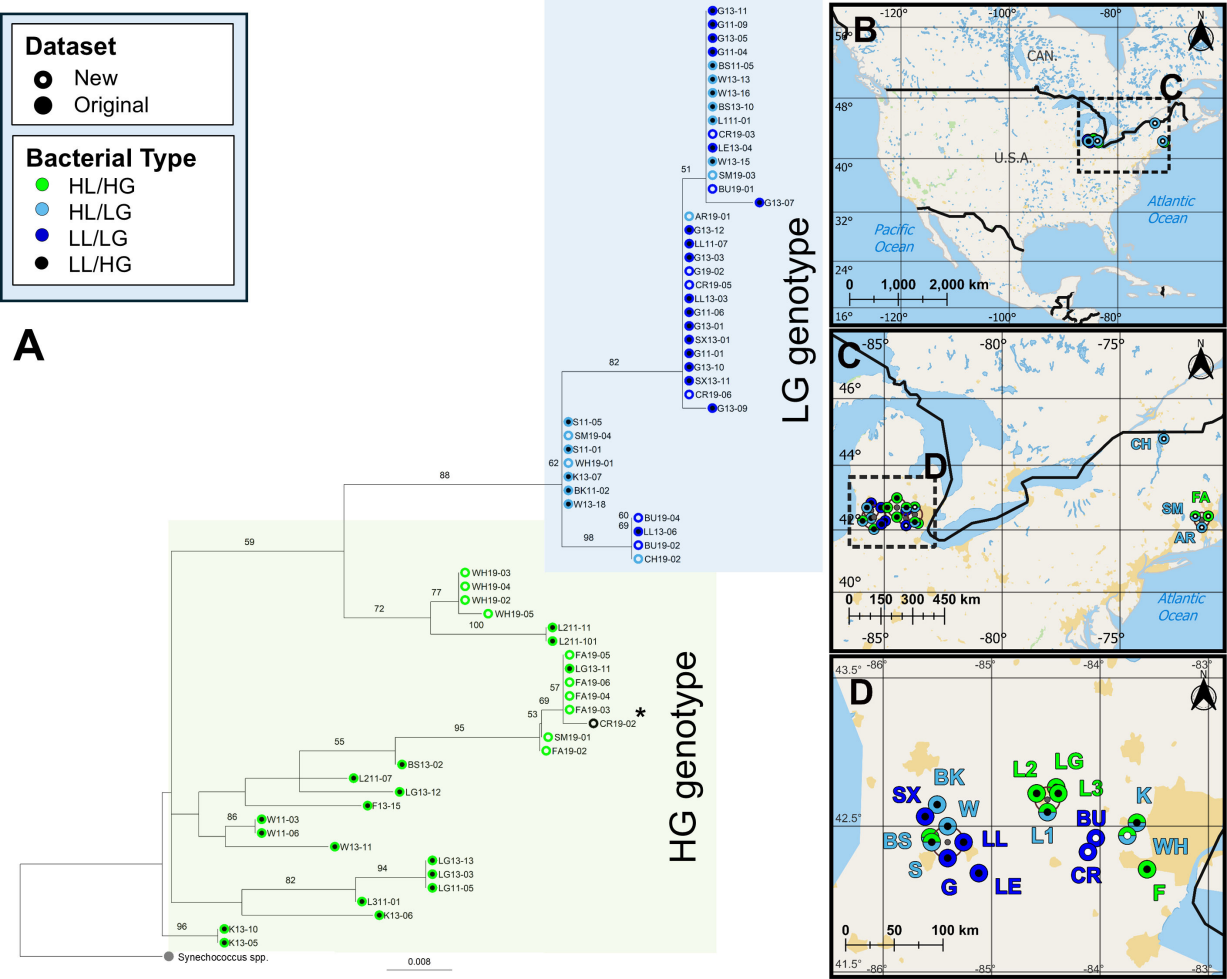
*(*A) Phylogenetic placement of 24 strains of *Microcystis aeruginosa* isolated in 2019 from lakes in the midwest and northeastern USA. These strains are nested within a larger phylogeny from our original dataset, isolated in 2011−2013 from an 8550 km^2^ region of MI, USA. Phylogeny based on the rRNA-ITSc region obtained via Sanger sequencing for 2019 strains and via extraction from metagenome assembled genomes for 2011−2013 strains. F19−02, G19−01 and CR19−01 excluded from phylogeny due to poor sequence quality. Placement of CR19−01, when included despite quality, indicated by asterisk. (B–D) Geographic origin of all strains. Filled dots indicate *M. aeruginosa* bacterial type, as determined by multi-locus sequence typing of metagenome-assembled genomes as described in [5]. Open dots indicate assigned bacterial type for 2019 isolates. Dark blue rings depict *M. aeruginosa* originating from oligotrophic lakes (LL/LG); light-blue depicts those from eutrophic and mesotrophic lakes that phylogenetically cluster with oligotrophic lakes (HL/LG type); green depicts all others from eutrophic and mesotrophic lakes (HL/HG); and half light-blue, half green rings (shown in maps) depict lakes where both HL/LG and HL/HG were isolated. See electronic supplementary material, table S1 for strain and lake metadata. HL, high-nutrient lake; HG, high-nutrient genotype; LG, low-nutrient genotype; LL, low-nutrient lake.

We found LL/LG and HL/LG types previously described from oligotrophic lakes within an 8550 km^2^ region in Michigan span a wider geographic range across the midwest and northeastern USA (figure 1; electronic supplementary material, figure S2). We then used 20 strains in a warming experiment, including seven assigned to HL/HG, five to HL/LG and seven to LL/LG. The final strain, belonging to a bacterial type not found in our prior work as it had an HG genotype but originated from an oligotrophic lake, was excluded from further analyses.

**Figure 2.**
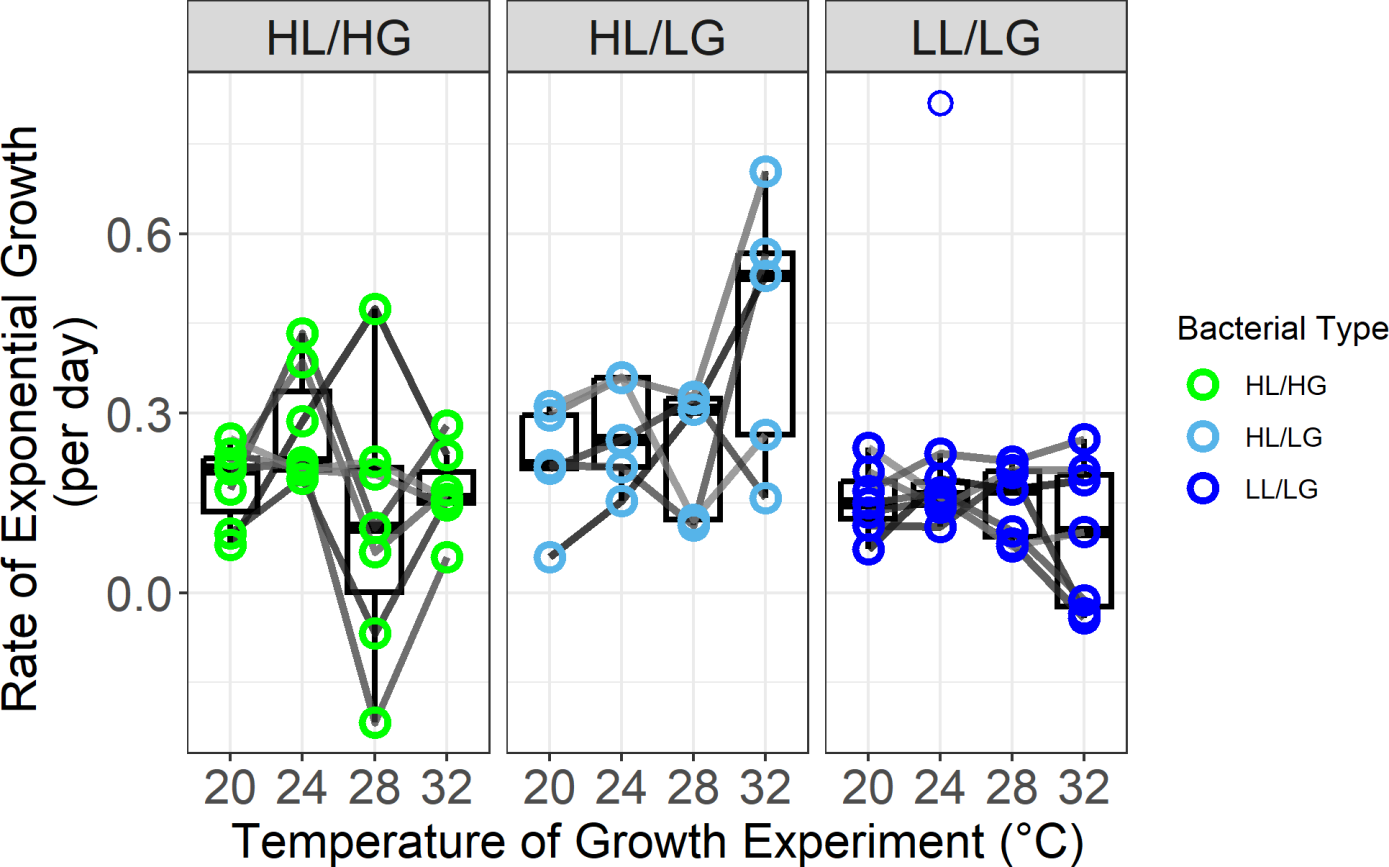
Exponential growth rates of oligotrophic types of *Microcystis aeruginosa*, HL/LG (5 strains) and LL/LG (7 strains), were, respectively, elevated or less negatively affected under warmer temperatures than the eutrophic HL/HG type (7 strains). Lines connect measurements of each strain grown across four temperatures. LME model showed a significant effect of type (*p* < 0.05), and a weaker effect of temperature and their interaction (*p* < 0.08). Further, a GAM with a type-specific responses to temperature fitted the data significantly better than a global response GAM. Note, the LL/LG outlier at 24°C is omitted from the strain’s trendline but retained in statistical analysis. HL, high-nutrient lake; HG, high-nutrient genotype; LG, low-nutrient genotype; LL, low-nutrient lake.

We first evaluated *M. aeruginosa* growth patterns during exponential growth in Week 1 of the warming study. We found that exponential growth varied by bacterial type, with a trend of type-specific responses to temperature (figure 2; linear mixed-effects (LME) model: type *F*_2_,_16_ = 164.16, *p* = 0.035, *η*^2^*p* = 0.34; temperature *F*_3,48_ = 2.55, *p* = 0.067, *η*^2^*p* = 0.14; type × temperature *F*_6,48_ = 2.070, *p* = 0.074, *η*^2^*p* = 0.21). Similarly, we found strong support for different responses to temperature by each type when using a hierarchical general additive model (GAM) framework, which does not have an assumption of linearity (electronic supplementary material, table S2, GAM: type-specific trendlines *p* = 0.001, *R*^2^ = 27.9%). We found that most strains grew with similar or higher growth rates at 24°C compared with 20°C: only two of seven HL/HG strains (28.6%), zero of five HL/LG strains (0%) and one of seven LL/LG (14.3%) had lower growth rates at 24°C than 20°C with median increases in growth rate from 20°C to 24°C for persisting strains of +78.2, +20.8 and+16.3%, respectively. In contrast, at higher temperatures, LG strains were less negatively affected by temperature than HL/HG strains, specifically, six of seven HL/HG strains (85.7%) had lower growth rates for 28°C compared with 20°C, while HL/LG had two of five (40%) and LL/LG had three of seven (42.9%) with median differences in growth rate from 20°C to 28°C for persisting strains of −15.0, +10.4 and +1.4%, respectively. At the highest temperature, all bacterial types had several strains negatively affected by temperature, as four of seven HL/HG (57.1%), two of five HL/LG (40%) and four of seven LL/LG strains (57.1%) showed lower growth rates at 32°C when compared with 20°C, but overall LG strains were less negatively affected with a median difference in growth rate from 20°C to 32°C for persisting strains of HL/LG at +137%, LL/LG at +26.1% and HL/HG at −20.4%.

**Figure 3.**
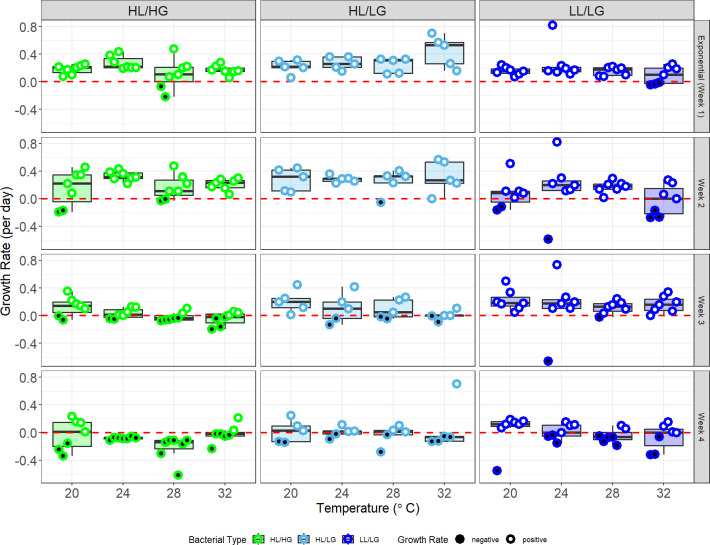
Bacterial type predicts growth rates and population persistence of *Microcystis aeruginosa* (see growth curves in electronic supplementary material, figure S3). Negative growth rates (i.e. population decline) shown with filled black data points and positive growth rates (i.e. population persistence) shown with open white points. Points spread on the *x*-axis for visibility but have no *y*-axis value alteration. LME model indicated significant effect of type (*p* < 0.01) and temperature (*p* < 0.05) on growth rates. A GAM with type-specific responses to temperature performed significantly better than a global response GAM. A cumulative link mixed-effect model for population persistence with strain and time point as random effects showed a significant effect of type and temperature. GAM, general additive model; HL, high-nutrient lake; HG, high-nutrient genotype; LG, low-nutrient genotype; LL, low-nutrient lake.

Upon evaluating growth patterns across the entire four week study, we find that growth was again context dependent on bacterial type (figure 3; electronic supplementary material, figure S3; LME with week as random effect: type *F*_2,70_ = 5.03, *p* = 0.009, η^2^*p* = 0.13; temperature *F*_3,219_ = 2.79, *p* = 0.042, η^2^*p* = 0.04; type × temperature *F*_6,219_ = 1.26, *p* = 0.28; see electronic supplementary material, figure S3 for LME with week as fixed effect). Furthermore, we find strong support for type-specific responses to temperature when dropping assumptions of linearity (electronic supplementary material, table S2, GAM: type-specific trendlines *p* = 0.008, *R*^2^ = 23.7%; see electronic supplementary material, figure S3 for GAM with week as a fixed effect). In particular, HL/LG strains tended to be less negatively affected by higher temperatures. However, all three types show marked decline by Week 4, by which time inorganic nutrients may have become scarce. Specifically, HL/LG has the highest average growth rate across all temperatures and weeks (Tukey’s tests: HL/HG versus HL/LG *p* = 0.008, HL/HG versus LL/LG *p* = 0.760, HL/LG versus LL/LG *p* = 0.046). Additionally, while growth rates across the four weeks do not show significant differences between bacterial types for 20°C and 24°C, there is a trend of higher growth rates for HL/LG versus HL/HG strains at 28°C and 32°C, with HL/LG also differing from LL/LG at 32°C (Tukey’s tests: 28°C HL/LG versus HL/HG *p* = 0.05; 32°C HL/LG versus HL/HG *p* = 0.035, HL/LG versus LL/LG *p* = 0.006; all other comparisons: *p* > 0.100).

By Week 4, all bacterial types had multiple strains starting to decline across all temperatures, but LG strains showed overall greater persistence. Across all temperatures, we found positive growth rates in 6 of 28 HL/HG populations (21.4%), 10 of 20 HL/LG populations (50%), and 19 of 28 LL/LG populations (67.9%). We note that in Week 1, all HL/HG strains show positive growth, while three LL/LG strains show negative growth. However, we previously found that LL/LG strains generally grow more slowly, which coupled with low cell densities at the start of the experiment, may have been more prone to measurement errors that indicated negative growth while the more rapidly growing HL/HG strains show clear early growth followed by rapid decline in the following weeks.

Further, population persistence (defined as holding a positive growth rate) over the four week experiment showed a strong dependence on both temperature and bacterial type (figure 3; CLMM: type: *p* < 0.01, temperature: *p* < 0.01, type × temperature: *p* = 0.45). Specifically, population persistence of HL/HG was notably lower at warmer temperatures, whereas a higher percentage of populations of the oligotrophic types persisted through the end of the study. For example, by Week 3, only 37.5% (four of seven) of HL/HG populations growing at 32°C showed positive growth compared with 100% (seven of seven) of LL/LG populations. We see similar results when comparing LG versus HG genotypes at 28°C, with three HG strains (28.6%) showing negative growth rates at Week 2, five (71.4%) by Week 3 and all (100%) strains no longer persisting by Week 4, while only one LG strain (7.7%) is non-persisting by Week 2, three (23.1%) by Week 3 and only seven (53.8%) by Week 4.

Next, we ran separate models for each bacterial type. The HL/HG type showed an effect of temperature on growth rates averaged across the four week experiment (HL/HG: temperature: *F*_3,81_ = 4.28, *p* = 0.007, *η*^2^*p* = 0.14). Specifically, we found that HL/HG growth at 28°C was slower than the growth at both 20°C and 24°C, while the growth rate at 32°C did not differ significantly from the other temperatures (Tukey’s tests: 28°C versus 20 °C *p* < 0.05, 28°C versus 24°C *p* < 0.05, all other comparisons *p* > 0.10). In contrast, the two oligotrophic types were not affected by temperature (HL/LG: temperature: *F*_3,57_ = 0.626, *p* = 0.601, *η*^2^*p* = 0.03; LL/LG: temperature: *F*_3,81_ = 1.55, *p* = 0.208, *η*^2^*p* = 0.05).

**Figure 4.**
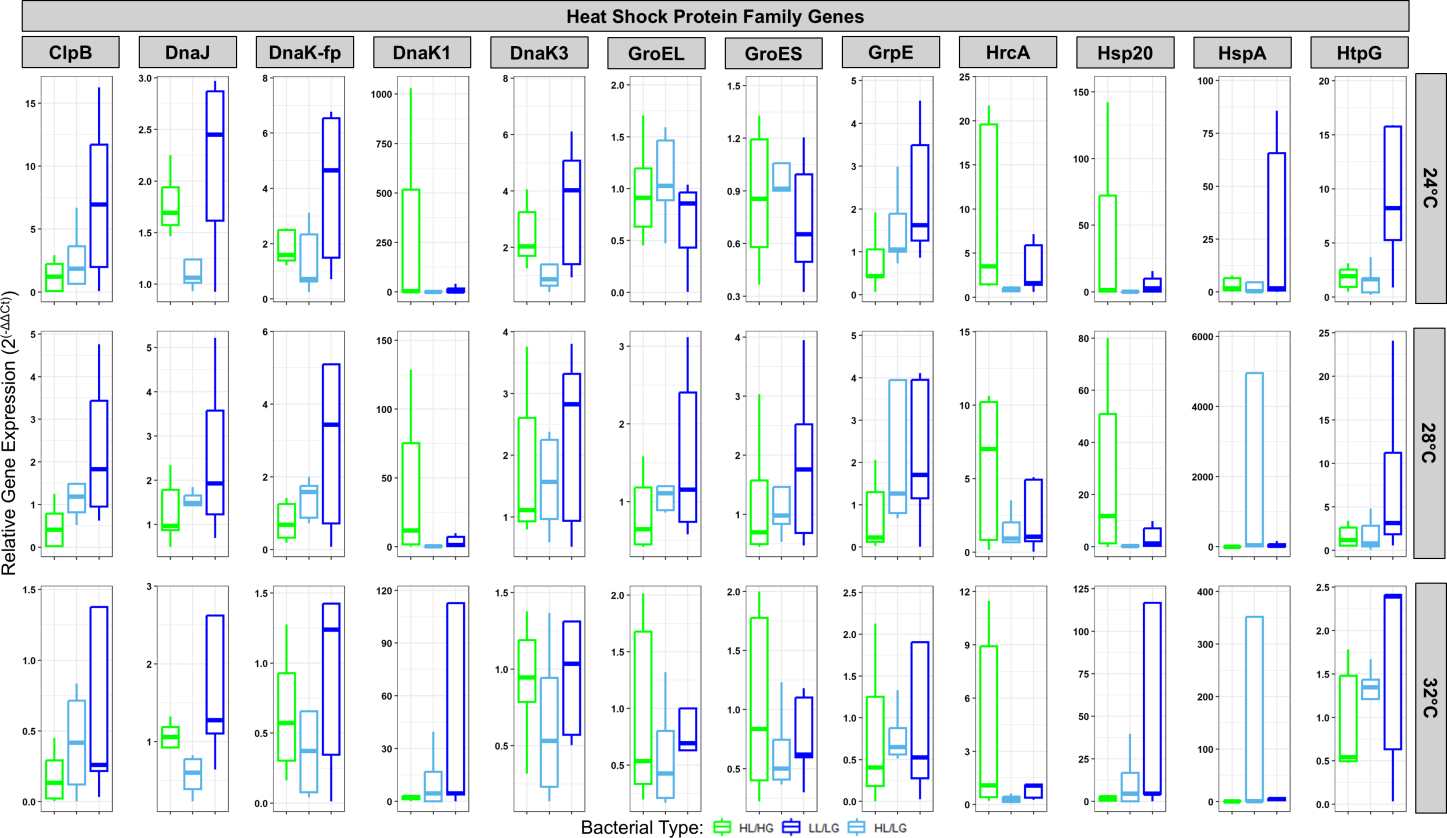
Bacterial type-specific responses to temperature of *Microcystis aeruginosa* through differential expression of genes encoding for heat shock. Type-specific responses to temperature were also observed for two models run on individual gene targets DnaK1 and Hsp20. Relative gene expression calculated as 2^(-ΔΔCt)^, with *rpoA* as the reference gene and 20°C as the reference condition. For visualization, each faceted plot has its own *y*-axis. See electronic supplementary material, figure S5 for the same data highlighting within-type responses to temperature and electronic supplementary material, figure S6 for normalized rather than raw data, with individual data points shown.

Given that these results suggest that climate warming may have differing effects on oligotrophic versus eutrophic types of *M. aeruginosa*, we aimed to further understand this phenomenon by evaluating the genetic content and gene expression responses of each bacterial type under warming scenarios. Surveying shotgun metagenomic datasets of previously collected *M. aeruginosa*, we found strain-level variation in copy number for genes involved in regulating thermotolerance (electronic supplementary material, figure S4). To directly measure thermotolerance as a result of both differential gene copy number and expression, we first tested for a widespread response of HSP family gene targets by including all measured HSPs in a single model. Here, we found that the effects of temperature on gene expression were highly bacterial type-specific (figure 4; electronic supplementary material, figures S5 and S6; analysis of variance (ANOVA): type: *F*_2,16_ = 2.17, *p* = 0.12, *η*^2^*p* = 0.02; temperature: *F*_2,402_ = 10.02, *p* < 0.01, *η*^2^*p* = 0.05; type × temperature: *F*_4,402_ = 3.67, *p* < 0.01, *η*^2^*p* = 0.04); however, we note that a large percentage of the variance in gene expression was not explained by our model, as indicated by the effect sizes. Most notably, the LL/LG type, whose growth rates were largely unaffected by temperature, showed markedly elevated expression of HSPs at warmer temperatures in comparison to other bacterial types (figure 4; electronic supplementary materials, figure S6). LL/LG had the highest mean relative gene expression in 58.3% of all 36 gene-by-temperature combinations and, notably, the highest expression in 75% of all 12 combinations within the 32°C treatment (figure 4). Further, the HL/LG type most often exhibited intermediate expression between the other two types. This ordered pattern of expression (i.e. HL/HG < HL/LG < LL/LG) was evident at warmer temperatures, including 46% of all 24 gene-by-temperature combinations at 28°C and 32°C (figure 4).

Given that constitutive versus inducible forms of HSPs may respond differently to thermal stress, we next focused on each target gene individually. We found that HSP expression declined with temperature, as would be expected for constitutively expressed proteins, including chaperones, for six different HSP targets (electronic supplementary material, figure S5; ANOVAs with fixed effect of temperature for ClpB: *F*_2,28_ = 7.41, *p* < 0.01, *η*^2^*p* = 0.35; DnaJ: *F*_2,28_ = 12.66, *p* < 0.01, *η*^2^*p* = 0.47; DnaK-fp: *F*_2,28_ = 8.20, *p* < 0.01, *η*^2^*p* = 0.37; DnaK3: *F*_2,28_ = 17.55, *p* < 0.01, *η*^2^*p* = 0.56; HrcA: *F*_2,28_ = 8.23, *p* < 0.01, *η*^2^*p* = 0.37; HtpG: *F*_2,28_ = 3.47, *p* = 0.045, η^2^*p* = 0.20). Toxin gene expression also declined with temperature for each bacterial type (electronic supplementary material, figure S7; *mcyE: F*_2,28_ = 12.09, *p* < 0.01, *η*^2^*p* = 0.46). However, in sharp contrast to this decline in expression with temperature, two HSPs were upregulated among oligotrophic types at warmer temperatures (electronic supplementary material, figure S5; ANOVA type × temperature for DnaK1: *F*_4,28_ = 2.83, *p* = 0.044, *η*^2^*p* = 0.29 and Hsp20: *F*_4,28_ = 2.90, *p* = 0.040, *η*^2^*p* = 0.29). Further, our results suggest a more finally tuned response to temperature increase in LG versus HG genotypes. Specifically, for LL/LG, five genes were differentially expressed between 24°C and 32°C (Tukey’s tests: DnaJ, DnaK-fp, DnaK3, MycE, Hsp20 and HtpG, all *p* < 0.05), and three were differentially expressed between 28°C and 32°C (MycE, DnaK1, and Hsp20, all *p* < 0.05). Similarly, for HL/LG, three genes were differentially expressed between 24°C and 32°C (Tukey’s tests: DnaJ, DnaK3, and HrcA, all *p* < 0.05), and four were differentially expressed between 28°C and 32°C (DnaJ, DnaK-fp, DnaK3 and HrcA, all *p* < 0.05). In contrast to these results for oligotrophic types, for HL/HG, we found less evidence of differential expression. Specifically, while four genes were differentially expressed between 24°C and 32°C (Tukey’s tests: DnaJ, DnaK3, MycE and HrcA, all *p* < 0.05), none were differentially expressed between 28°C and 32°C.

**Figure 5.**
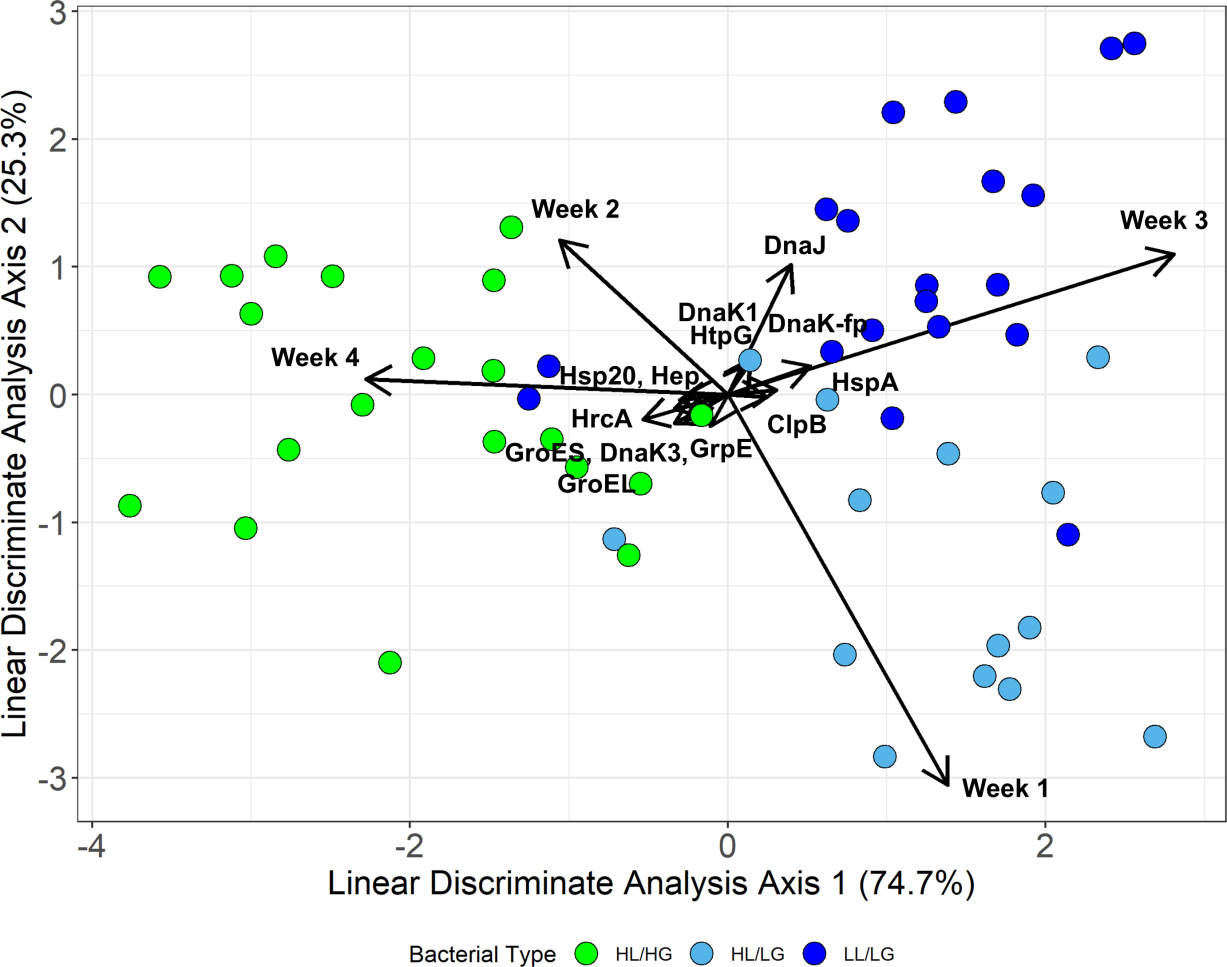
Bacterial types differ in overall patterns of growth and gene expression dynamics via linear discriminant analysis. Arrows indicate coefficients of variance explained by each variable (see electronic supplementary material, figure S8 for magnification of gene expression arrows). Data for all temperatures except 20°C are included in the model to allow for the calculation of relative gene expression with 20°C as the baseline. Week *x* refers to the growth rate during that week.

While we found clear variation between bacterial types, substantial variation within types in terms of both growth dynamics and gene expression is evident. We found that despite this intratype variation, bacterial type is a strong predictor of strain growth and expression rates (MANOVA: type *F*_34,58_ = 2.74, *p* < 0.001, *η*^2^*p* = 0.62; temperature *F*_34,58_ = 1.39, *p* = 0.131; type × temperature: *F*_68,124_ = 0.70, *p* = 0.950). Additionally, bacterial type remains a significant predictor when considering only growth dynamics or only gene expression rates in separate multivariate models (growth dynamics only model: type *F*_8,84_ = 2.24, *p* = 0.032, *η*^2^*p* = 0.18; temperature *F*_8,84_ = 1.87, *p* = 0.075, *η*^2^*p* = 0.15; type × temperature: *F*_16,176_ = 0.89, *p* = 0.584; Gene expression only model: type *F*_26,66_ = 3.40, *p* < 0.0001, *η*^2^*p* = 0.57; temperature *F*_26,66_ = 0.97, *p* = 0.520; type × temperature: *F*_52,140_ = 0.58, *p* = 0.99). We also found clear clustering by type using a linear discriminates analysis. Specifically, LG and HG strains separated along the first axis of discrimination, which explained 74.7% of the between-class variation and was most heavily weighted by Week 3 and 4 growth rates, and ClpB, DnaK3, DnaK-fp, GroES, GroEL, Hep, HrcA, HspA, and Hsp20 expression; while HL/LG and LL/LG strains separated along the second axis of discrimination, which explained 25.3% of the between-class variation and was most heavily weighted by differences in week 1 and 2 growth rates, and DnaK1, DnaJ, GrpE, and HtpG expression rates (figure 5; electronic supplementary material, figure S8 and table S3).

## 4. Discussion

*Microcystis aeruginosa* is the dominant cause of freshwater harmful algal blooms worldwide and can inhabit an impressive range of environments that span over 20-fold in phosphorus levels due in part to extensive genetic diversity [32–34]. We show that this intraspecific biodiversity within *M. aeruginosa* is essential for predicting how this cyanobacterium will respond to the warming climate [6]. Overall, our results suggest that the *M. aeruginosa* types show different patterns of growth in response to temperature, both during exponential growth and across a month of heat exposure. We find that oligotrophic-adapted bacterial types of *M. aeruginosa* are more likely to survive long term at higher temperatures, with their exponential rates of growth generally unaffected by or even benefitting from much warmer temperatures. In contrast, the classic eutrophic type of *M. aeruginosa* showed a notable decrease in growth rate and population persistence at higher temperatures, especially across longer timespans, suggesting that climate warming may cause strains of this type to experience a competitive disadvantage. Interestingly, the typically slower growing LL/LG was generally intermediate in growth rate between HL/LG and HL/HG at higher temperatures, suggesting that it survives better than HL/HG but lacks the rapid growth of HL/LG. The latter shows genomic markers for surviving oligotrophic conditions but may nonetheless be better equipped to utilize the higher nutrient environment of the mesotrophic and eutrophic lakes that these strains were originally isolated from.

Considering that not all strains of *M. aeruginosa* carry the *mcy* toxin operon whose product causes wildlife mortality and human illness, and that oligotrophic strains are more likely to carry functional *mcy* operons, our results suggest that future blooms that develop under climate warming may become more toxic and more tolerant of low-nutrient conditions [35]. This result is in-line with the growing evidence that cyanobacterial blooms are becoming more toxic through time [36,37]. However, the competitive dynamics in complex natural systems are multifactorial and challenging to predict from outcomes of controlled laboratory-based experiments, so additional investigation into whether oligotrophic strains will indeed become more prevalent in natural systems under climate warming should be further investigated. Furthermore, we acknowledge that, while our bacterial type groupings are supported by genomic evidence from our previous work (including a high-resolution phylogeny and genome-wide protein functional analyses), as well as a multivariate analysis in this study, our results nevertheless also highlight the level of between-strain variability found within these bacterial types [5]. Additional investigation to better parse and explain this inter- and intratype variation will be necessary to further expand and refine our understanding of these bacterial types of *M. aeruginosa*.

We previously documented the differential abundance of these three bacterial types of *M. aeruginosa* across a 20-fold gradient of TP in Michigan lakes [5]. Here, we show that this pattern of locally adapted bacterial types exists across a much larger region of lakes spanning the midwestern and eastern USA. Other cyanobacteria are also known to exhibit clades based on environmental conditions; for example, *Prochlorococcus* populations group into clades adapted to high- and low light intensity which occupy and exploit different depths of the euphotic zone in the ocean [38,39]. Furthermore, the major light intensity-adapted clades of *Prochlorococcus* are subdivided into further genomic subclades, several of which have evidence for adaptation to specific temperature ranges or/and nutrient conditions such as iron limitation [38,40]. For example, there is evidence that while the whole genome content within the HLII clade of *Prochlorococcus* is best explained by temperature, its phylogenetic subclades are most strongly linked to adaptation to P limitation [41]. The impressive diversity in these marine cyanobacteria is suggested to be a form of niche partitioning, allowing them to fill the many available microniches in the highly spatiotemporally variable ocean. In a similar manner, the bacterial types we previously described in *M. aeruginosa*, and find further evidence for in this study, are suggestive of adaptation to local niches in freshwater lakes, including both high and low phosphorus niches in eutrophic and mesotrophic lakes and the low phosphorus conditions of oligotrophic lakes [5]. As other cyanobacteria, such as *Prochlorococcus,* have been shown to have subclades adapted to differing temperature conditions within their major clades, it is conceivable that strains of *M. aeruginosa* grouped by our nutrient-limitation-based types may show considerable variation in their response to temperature. Indeed, our bacterial types do exhibit appreciable intratype variation. Still, with evidence of genome-wide functional similarity within types, such as adaptation to oligotrophic conditions among LG strains via genome streamlining and increased copies of phosphorus acquisition genes, paired with the clustering by bacterial type for growth and expression rate patterns that we observed within this study, our results suggest that for *M. aeruginosa* these types remain a useful and predictive means of categorization [5]. Nevertheless, future studies to better characterize intratype variation in temperature tolerance of *M. aeruginosa* clades will be essential to fully understand the complex environmental adaptation and future potential of *M. aeruginosa.*

While oligotrophic types have not yet been documented outside of North America, there is strong evidence that *M. aeruginosa* is capable of global dispersal. For example, early research on the biogeography of *M. aeruginosa* found no clear correlation between genetic and geographic distance, and although more recent research has identified patterns of genetic structure across spatial scales, the ability of *Microcystis* to disperse globally is well accepted [42–44]. Mechanisms of dispersal are thought to include aerosols, atmospheric bridges and both human- and wildlife-mediated dispersal [45–48]. Therefore, oligotrophic types might readily disperse into both inland waters previously uninhabited by *Microcystis* and those currently dominated by the eutrophic type. Such potentially rapid dispersal suggests that even if future environmental conditions caused by climate change and human activities cause mismatches between strains and lake conditions [34], these effects might be temporary with the rapid establishment of alternative bacterial types adapted to and capable of forming blooms under the new conditions.

To further investigate the mechanistic underpinnings of how each *M. aeruginosa* type tolerates warming, we analysed gene expression in the HSP gene family. In agreement with our growth rate and population persistence data, we found that the eutrophic and oligotrophic types responded differently in their expression patterns to warming. While all three types showed differential expression across the HSP gene family in response to warming, the magnitude of this heat shock response was substantially greater among oligotrophic types, as indicated by a significant type × temperature interaction in our statistical model. These results provide a mechanistic foundation for the observed higher rates of population persistence of these types, suggesting that as the climate warms, either strains of the HL/HG type will have to adapt or may be outcompeted by the oligotrophic-adapted types.

While we found that bacterial type-specific responses to temperature were widespread across the HSP gene family, we found that two genes in particular, DnaK1 and Hsp20, showed contrasting responses between the oligotrophic and eutrophic types. The DnaK gene family, or HSP70 family, is a ubiquitous family of highly conserved molecular chaperones containing both constitutively expressed and stress-inducible forms [21]. Hsp20s, which belong to the small HSP family, are known for their strong induction by a variety of heat stresses [49,50]. Protein expression, both of constitutive HSPs as well as a diverse range of other proteins, are often downregulated during thermal stress due to slowing of translation as a whole and prioritized production of inducible forms of HSPs that are key for organismal survival during heat stress [51]. Our results suggest that oligotrophic types are more thermotolerant than the eutrophic type by maintaining elevated levels of constitutively expressed HSPs at warmer temperatures, as well as by substantially upregulating inducible HSPs, including DnaK1 and HSP20. However, there also remains appreciable intratype variation in gene expression. As previously discussed, some other cyanobacterial clades have been shown to contain subclades adapted to different temperatures. Therefore, while our motivation was to investigate type level trends in this study, an important future direction will be to further investigate intratype variation in thermotolerance. In summary, in addition to elevated expression by oligotrophic types of *M. aeruginosa* compared with the eutrophic type across most of the tested gene targets within the HSP family, we find highly type-specific responses to temperature in key members of both large and small HSPs responsible for mediating the heat shock response.

Our results share some similarities with prior work on the expression of HSPs in *M. aeruginosa* that found the upregulation of *HspA* and *HtpG* in response to heat and cold shocks [28]. Our warming experiment found a significant effect of temperature on *HtpG* expression, although not *HspA*. However, it should be noted that we used an acclimation approach to our warming study rather than a temperature shock. This probably elicits a different physiological response with those HSPs that respond rapidly to heat spikes typically different from those that respond to persistent high temperatures over long time periods.

We also found that temperature had a negative effect on the expression of *mcyE*, which is indicative of microcystin production in approximately 80% of cases [31]. However, our result contrasts with a recent meta-analysis that found a positive correlation between temperature and microcystin [36]. Additionally, we did not find a significant effect of bacterial type on the differential expression of *mcyE*. Based on prior work that had found the functional *mcy* operon to be more prevalent among oligotrophic types, we hypothesized that we might find greater expression responses of *mcyE* among these types [35]. While we did not find evidence to support this, nor the expected positive correlation between *mcy* operon gene expression and temperature, it is possible other conditions maintained during the study were not conducive to toxin production. For example, while the specific conditions that induce microcystin production are still debated, high-cell density and nutrient limitation are thought to be two important factors [52–55]. It is, therefore, conceivable that toxin production may have been relatively low either due to the lower cell densities in culture (thereby preventing activation of potential mechanisms for cell-density-dependent increased toxin production) or possible development of N limitation in month-old cultures (microcystin is an N-rich compound). It is also possible that we did not find differences in *mcyE* expression among bacterial types due to strain-by-strain variation within types, as others have found variation in microcystin production even between closely related strains [35]. Therefore, our lack of evidence for an effect of bacterial type on *mcyE* expression may be due to the complexity of *mcy* operon regulation.

There are several potential explanations for why oligotrophic types may be more thermotolerant. First, there is a precedence for a connection between genome streamlining and thermotolerance. Streamlining is a strategy involving both cellular and genome downsizing to allow more efficient use of sparse nutrients, with important ecological implications [56]. In thermophilic bacteria, both the size of the genome and the percentage of intergenic regions were found to be negatively correlated with the temperature at which the bacterial strain was found [57]. Similarly, we previously reported that LL/LG and HL/LG genomes contained a higher percentage of coding versus non-coding DNA, and therefore we could infer from Sabath *et al*. that these oligotrophic types might tolerate higher temperatures, as we do in fact find in the present study [5]. However, the authors hypothesized that the correlation was due to indirect selection for genome streamlining, driven by direct selection for smaller cell size at higher temperatures, as genome size is thought to constrain cell size. Yet, while we found multiple indicators of genome streamlining in the LL/LG type of *M. aeruginosa*, we did not find evidence for reduced genome size [5]. Furthermore, as *Microcystis* forms colonies that are frequently composed of 10^4^−10^5^ cells, the functional implications of cell size versus colony size on temperature tolerance would need to be considered.

Another potential explanation for why oligotrophic types may be more thermotolerant is the co-occurrence of two notable selective pressures within cyanobacterial blooms. Late-stage blooms are typically characterized by both the depletion of bioavailable nutrients and warmer temperatures [58]. Such blooms typically develop later into the summer when atmospheric temperatures are higher [59,60]. Additionally, large cyanobacterial blooms are themselves heat traps, causing elevated local surface water temperatures [13]. Therefore, the streamlining of oligotrophic types may provide a selective advantage when exposed to two stressors that often occur simultaneously in late-stage blooms.

Last, an additional explanation for why oligotrophic types may be more thermotolerant stems from redundancy in cellular stress responses. While organisms inhabiting warmer environments are well known to have higher temperature thresholds for the induction of HSP expression, there is also evidence from a range of systems that populations adapted to extreme environments characterized by conditions other than warm temperatures also have an increased basal level of HSPs [23]. This can be explained by the fact that HSPs can be thought of as a more general response to stress overall [22,23]. For example, increased sodium levels in soils, which can cause osmotic stress, have been found to induce HSP expression in grasses [61]. Further, there is evidence that caloric limitation in eukaryotes can increase HSP responses [62]. Similarly, comparative work of two species within the unicellular green algal genus *Chlamydomonas* found that *Chlamydomonas acidophila,* which is adapted to acidic environments, exhibited significantly higher levels of HSPs under control conditions compared with the more generalist *Chlamydomonas reinhardtii* [63]. Overall, it is conceivable that by evolving to a nutrient-limited environment, the LL/LG type may be more fine-tuned to respond to multiple types of stress or may be primed with higher constitutive levels of HSPs. A potential complication to this interpretation is genome streamlining within the LL/LG type, which may affect the number and expression of HSP family genes. Streamlined bacteria tend to have fewer sigma factors, which initiate transcription, and so could include a loss of sigma factors targeting HSPs and complex control of HSP expression. Ultimately, further research may elucidate what effects genome streamlining in bacteria may have on the regulation of HSPs. Nevertheless, the consistent growth rates that we observed in the LL/LG type across temperatures may be due to elevated constitutive levels of HSPs that are sufficient to mitigate any effects of temperature in laboratory cultures.

There are several important limitations to our study. First, our strains were not axenic (i.e. not free of heterotrophic bacteria) and so it is conceivable some results may differ with variation in the composition of the *M. aeruginosa* microbiome. It is also possible that some of the between strain variation we observe within bacterial types is due to differences in microbiome compositions between the strains. For example, our recent work found that *M. aeruginosa* has greater fitness and competitive ability when xenic [64]. Furthermore, we recently found that heterotrophic bacteria inhabiting the microbiome of oligotrophic types of *M. aeruginosa* share many of the same indicators of genome streamlining and features that facilitate survival in nutrient-depleted environments as had been found in their cyanobacterial hosts [26]. Therefore, differences in thermotolerance may also exist between the microbiomes of our *M. aeruginosa* types. Further, the complexity of natural systems is also expected to affect HSP expression. For example, heat shock response has been shown to be affected by light in some cyanobacteria [65]. Nevertheless, with increasing temperatures being the key feature of anthropogenic climate change, our results, which show responses to increased temperature with all other factors kept constant, should still provide valuable insight for predicting the effects of climate change on *Microcystis* and harmful bloom dynamics. Further, while not all inland waters are expected to reach the highest temperature tested in our study (32°C), one of the largest and deepest *Microcystis*-source lakes used in our study, oligotrophic Gull Lake (MI, USA), reached a 24 h epilimnetic mean temperature of 30°C as far back as 2012 [34]. Shallow eutrophic lakes, where *Microcystis* is more commonly found, have less thermal inertia and are likely to more readily achieve these extreme temperatures in the near future. Given the global distribution of *Microcystis*, these experimental temperatures are also highly applicable to subtropical and tropical environments. Finally, we note that a limitation to our study and classification system is the large variation that still exists between strains within bacterial types and our single biological replicate per strain per temperature condition. While these three types are well supported by genomic analysis in our previously published work, as well as the trait data in this study that shows significant clustering by bacterial type, future research should further consider and attempt to explain this intratype variability. For example, an expanded study with biological replication within strain could further clarify strain-by strain variation in temperature tolerance within bacterial types.

In conclusion, our results show that strains adapted to oligotrophy are better equipped to persist during warming scenarios, with higher or unchanged growth rates when comparing low to high temperature conditions. In contrast, strains adapted to eutrophic conditions tend to have lower persistence at higher temperatures. We fortify these results with gene expression data showing that different types of *M. aeruginosa* have differing patterns of expression of HSP genes in response to increased temperatures. Given previous research suggesting that oligotrophic strains are more likely to produce microcystin, a dangerous toxin, our results suggests that climate warming may select for the formation of cyanobacterial blooms with enhanced capacity to both produce the microcystin toxin and tolerate nutrient limitation. Therefore, intraspecific genetic diversity within *M. aeruginosa* may be key in predicting the dynamic of freshwater harmful algal blooms under climate warming.

## Data Availability

Metadata are archived in Dryad [66]. Sequences used for phylogenies are available at NCBI PQ666794-PQ666862. Supplementary material is available online [67].

## References

[1] Crutsinger GM, Collins MD, Fordyce JA, Gompert Z, Nice CC, Sanders NJ. 2006 Plant genotypic diversity predicts community structure and governs an ecosystem process. Science **313**, 966–968. (10.1126/science.1128326) 16917062

[2] Whitham TG *et al*. 2006 A framework for community and ecosystem genetics: from genes to ecosystems. Nat. Rev. Genet. **7**, 510–523. (10.1038/nrg1877) 16778835

[3] Schopf JW. 2012 The fossil record of Cyanobacteria. In Ecology of Cyanobacteria II: their diversity in space and time (ed. BA Whitton), pp. 15–36. Dordrecht, The Netherlands: Springer. (10.1007/978-94-007-3855-3_2)

[4] Whitton BA. 2012 Ecology of Cyanobacteria II: their diversity in space and time. Dordrecht, The Netherlands: Springer.

[5] Jackrel SL, White JD, Evans JT, Buffin K, Hayden K, Sarnelle O, Denef VJ. 2019 Genome evolution and host‐microbiome shifts correspond with intraspecific niche divergence within harmful algal bloom‐forming Microcystis aeruginosa. Mol. Ecol. **28**, 3994–4011. (10.1111/mec.15198) 31344288

[6] Huisman J, Codd GA, Paerl HW, Ibelings BW, Verspagen JMH, Visser PM. 2018 Cyanobacterial blooms. Nat. Rev. Microbiol. **16**, 471–483. (10.1038/s41579-018-0040-1) 29946124

[7] Hamilton DP, Wood SA, Dietrich DR, Puddick J. 2014 Costs of harmful blooms of freshwater cyanobacteria. In Cyanobacteria, pp. 245–256. John Wiley & Sons,Ltd. (10.1002/9781118402238)

[8] Paerl HW, Huisman J. 2009 Climate change: a catalyst for global expansion of harmful cyanobacterial blooms. Environ. Microbiol. Rep. **1**, 27–37. (10.1111/j.1758-2229.2008.00004.x) 23765717

[9] Jöhnk KD, Huisman J, Sharples J, Sommeijer B, Visser PM, Stroom JM. 2008 Summer heatwaves promote blooms of harmful cyanobacteria. Glob. Chang. Biol. **14**, 495–512. (10.1111/j.1365-2486.2007.01510.x)

[10] Coles JF, Jones RC. 2000 Effect of temperature on photosynthesis‐light response and growth of four phytoplankton species isolated from a tidal freshwater river. J. Phycol. **36**, 7–16. (10.1046/j.1529-8817.2000.98219.x)

[11] Robarts RD, Zohary T. 1987 Temperature effects on photosynthetic capacity, respiration, and growth rates of bloom‐forming cyanobacteria. N. Z. J. Mar. Freshw. Res. **21**, 391–399. (10.1080/00288330.1987.9516235)

[12] Hense I. 2007 Regulative feedback mechanisms in cyanobacteria-driven systems: a model study. Mar. Ecol. Prog. Ser. **339**, 41–47. (10.3354/meps339041)

[13] Kahru M, Leppanen JM, Rud O. 1993 Cyanobacterial blooms cause heating of the sea surface. Mar. Ecol. Prog. Ser. **101**, 1–7. (10.3354/meps101001)

[14] Harke MJ, Steffen MM, Gobler CJ, Otten TG, Wilhelm SW, Wood SA, Paerl HW. 2016 A review of the global ecology, genomics, and biogeography of the toxic cyanobacterium, Microcystis spp. Harmful Algae **54**, 4–20. (10.1016/j.hal.2015.12.007) 28073480

[15] Masango MG, Myburgh JG, Labuschagne L, Govender D, Bengis RG, Naicker D. 2010 Assessment of Microcystis bloom toxicity associated with wildlife mortality in the Kruger National Park, South Africa. J. Wildl. Dis. **46**, 95–102. (10.7589/0090-3558-46.1.95) 20090022

[16] Steffen MM *et al*. 2017 Ecophysiological examination of the Lake Erie Microcystis bloom in 2014: linkages between biology and the water supply shutdown of Toledo, OH. Environ. Sci. Technol. **51**, 6745–6755. (10.1021/acs.est.7b00856) 28535339

[17] Qin B, Zhu G, Gao G, Zhang Y, Li W, Paerl HW, Carmichael WW. 2010 A drinking water crisis in Lake Taihu, China: linkage to climatic variability and lake management. Environ. Manag. **45**, 105–112. (10.1007/s00267-009-9393-6) 19915899

[18] Wetzel RG. 2001 Limnology: lake and river ecosystems. San Diego, CA: Academic Press.

[19] Scavia D *et al*. 2014 Assessing and addressing the re-eutrophication of Lake Erie: central basin hypoxia. J. Gt. Lakes Res. **40**, 226–246.

[20] Parsell DA, Lindquist S. 1993 The function of heat-shock proteins in stress tolerance: degradation and reactivation of damaged proteins. Annu. Rev. Genet. **27**, 437–496. (10.1146/annurev.ge.27.120193.002253) 8122909

[21] Hartl FU, Bracher A, Hayer-Hartl M. 2011 Molecular chaperones in protein folding and proteostasis. Nature **475**, 324–332. (10.1038/nature10317) 21776078

[22] Lindquist S. 1986 The heat-shock response. Annu. Rev. Biochem. **55**, 1151–1191. (10.1146/annurev.bi.55.070186.005443) 2427013

[23] Feder ME, Hofmann GE. 1999 Heat-shock proteins, molecular chaperones, and the stress response: evolutionary and ecological physiology. Annu. Rev. Physiol. **61**, 243–282. (10.1146/annurev.physiol.61.1.243) 10099689

[24] White JD, Kaul RB, Knoll LB, Wilson AE, Sarnelle O. 2011 Large variation in vulnerability to grazing within a population of the colonial phytoplankter, Microcystis aeruginosa. Limnol. Oceanogr. **56**, 1714–1724. (10.4319/lo.2011.56.5.1714)

[25] Stemberger RS. 1981 A general approach to the culture of planktonic rotifers. Can. J. Fish. Aquat. Sci. **38**, 721–724. (10.1139/f81-095)

[26] Jackrel SL, White JD, Perez-Coronel E, Koch RY. 2023 Selection for oligotrophy among bacteria inhabiting host microbiomes. mBio **14**, e0141523. (10.1128/mbio.01415-23) 37646528 PMC10653850

[27] Fortunato CS, Huber JA. 2016 Coupled RNA-SIP and metatranscriptomics of active chemolithoautotrophic communities at a deep-sea hydrothermal vent. ISME J. **10**, 1925–1938. (10.1038/ismej.2015.258) 26872039 PMC5029171

[28] Rhee JS, Ki JS, Kim BM, Hwang SJ, Choi IY, Lee JS. 2012 HspA and HtpG enhance thermotolerance in the cyanobacterium, Microcystis aeruginosa NIES-298. J. Microbiol. Biotechnol. **22**, 118–125. (10.4014/jmb.1108.08001) 22297228

[29] Jungblut AD, Neilan BA. 2006 Molecular identification and evolution of the cyclic peptide hepatotoxins, microcystin and nodularin, synthetase genes in three orders of Cyanobacteria. Arch. Microbiol. **185**, 107–114. (10.1007/s00203-005-0073-5) 16402223

[30] Lu J, Struewing I, Wymer L, Tettenhorst DR, Shoemaker J, Allen J. 2020 Use of qPCR and RT-qPCR for monitoring variations of microcystin producers and as an early warning system to predict toxin production in an Ohio inland lake. Water Res. **170**, 115262. (10.1016/j.watres.2019.115262) 31785564 PMC7075668

[31] Pacheco A, Guedes I, Azevedo S. 2016 Is qPCR a reliable indicator of cyanotoxin risk in freshwater? Toxins **8**, 172. (10.3390/toxins8060172) 27338471 PMC4926139

[32] Cao H, Xu D, Zhang T, Ren Q, Xiang L, Ning C, Zhang Y, Gao R. 2022 Comprehensive and functional analyses reveal the genomic diversity and potential toxicity of Microcystis. Harmful Algae **113**, 102186. (10.1016/j.hal.2022.102186) 35287927

[33] Humbert JF *et al*. 2013 A tribute to disorder in the genome of the bloom-forming freshwater cyanobacterium Microcystis aeruginosa. PLoS ONE **8**, e70747. (10.1371/journal.pone.0070747) 23950996 PMC3741299

[34] White JD, Sarnelle O, Hamilton SK. 2017 Unexpected population response to increasing temperature in the context of a strong species interaction. Ecol. Appl. **27**, 1657–1665. (10.1002/eap.1558) 28401624

[35] Berry MA, White JD, Davis TW, Jain S, Johengen TH, Dick GJ, Sarnelle O, Denef VJ. 2017 Are oligotypes meaningful ecological and phylogenetic units? A case study of Microcystis in freshwater lakes. Front. Microbiol. **08**, 365. (10.3389/fmicb.2017.00365) PMC5341627 28337183

[36] Buley RP, Gladfelter MF, Fernandez-Figueroa EG, Wilson AE. 2022 Can correlational analyses help determine the drivers of microcystin occurrence in freshwater ecosystems? A meta-analysis of microcystin and associated water quality parameters. Environ. Monit. Assess. **194**, 493. (10.1007/s10661-022-10114-8) 35690674

[37] Heathcote AJ, Taranu ZE, Tromas N, MacIntyre‐Newell M, Leavitt PR, Pick FR. 2023 Sedimentary DNA and pigments show increasing abundance and toxicity of cyanoHABs during the Anthropocene. Freshw. Biol. **68**, 1859–1874. (10.1111/fwb.14069)

[38] Biller SJ, Berube PM, Lindell D, Chisholm SW. 2015 Prochlorococcus: the structure and function of collective diversity. Nat. Rev. Microbiol. **13**, 13–27. (10.1038/nrmicro3378) 25435307

[39] Chen MY, Teng WK, Zhao L, Hu CX, Zhou YK, Han BP, Song LR, Shu WS. 2021 Comparative genomics reveals insights into cyanobacterial evolution and habitat adaptation. ISME J. **15**, 211–227. (10.1038/s41396-020-00775-z) 32943748 PMC7852516

[40] Larkin AA, Blinebry SK, Howes C, Lin Y, Loftus SE, Schmaus CA, Zinser ER, Johnson ZI. 2016 Niche partitioning and biogeography of high light adapted Prochlorococcus across taxonomic ranks in the North Pacific. ISME J. **10**, 1555–1567. (10.1038/ismej.2015.244) 26800235 PMC4918451

[41] Ustick LJ, Larkin AA, Martiny AC. 2023 Global scale phylogeography of functional traits and microdiversity in Prochlorococcus. ISME J. **17**, 1671–1679. (10.1038/s41396-023-01469-y) 37454234 PMC10504305

[42] van Gremberghe I, Leliaert F, Mergeay J, Vanormelingen P, Van der Gucht K, Debeer AE, Lacerot G, De Meester L, Vyverman W. 2011 Lack of phylogeographic structure in the freshwater cyanobacterium Microcystis aeruginosa suggests global dispersal. PLoS One **6**, e19561. (10.1371/journal.pone.0019561) 21573169 PMC3088681

[43] Moreira C, Spillane C, Fathalli A, Vasconcelos V, Antunes A. 2014 African origin and Europe-mediated global dispersal of the cyanobacterium Microcystis aeruginosa. Curr. Microbiol. **69**, 628–633. (10.1007/s00284-014-0628-2) 24952206

[44] Shirani S, Hellweger FL. 2017 Neutral evolution and dispersal limitation produce biogeographic patterns in Microcystis aeruginosa populations of lake systems. Microb. Ecol. **74**, 416–426. (10.1007/s00248-017-0963-5) 28303312

[45] Sharma NK, Singh S. 2010 Differential aerosolization of algal and cyanobacterial particles in the atmosphere. Indian J. Microbiol. **50**, 468–473. (10.1007/s12088-011-0146-x) 22282617 PMC3209841

[46] Lewandowska AU, Śliwińska-Wilczewska S, Woźniczka D. 2017 Identification of cyanobacteria and microalgae in aerosols of various sizes in the air over the southern Baltic Sea. Mar. Pollut. Bull. **125**, 30–38. (10.1016/j.marpolbul.2017.07.064) 28823424

[47] Doblin MA, Coyne KJ, Rinta-Kanto JM, Wilhelm SW, Dobbs FC. 2007 Dynamics and short-term survival of toxic cyanobacteria species in ballast water from NOBOB vessels transiting the Great Lakes—implications for HAB invasions. Harmful Algae **6**, 519–530. (10.1016/j.hal.2006.05.007)

[48] Curren E, Leong SCY. 2020 Natural and anthropogenic dispersal of cyanobacteria: a review. Hydrobiologia **847**, 2801–2822. (10.1007/s10750-020-04286-y)

[49] Basha E, O’Neill H, Vierling E. 2012 Small heat shock proteins and α-crystallins: dynamic proteins with flexible functions. Trends Biochem. Sci. **37**, 106–117. (10.1016/j.tibs.2011.11.005) 22177323 PMC3460807

[50] Srivastava AK, Rai AN, Neilan BA. 2013 Stress biology of cyanobacteria: molecular mechanisms to cellular responses. Boca Raton, FL: CRC Press.

[51] Klaips CL, Jayaraj GG, Hartl FU. 2018 Pathways of cellular proteostasis in aging and disease. J. Cell Biol. **217**, 51–63. (10.1083/jcb.201709072) 29127110 PMC5748993

[52] Horst GP, Sarnelle O, White JD, Hamilton SK, Kaul RB, Bressie JD. 2014 Nitrogen availability increases the toxin quota of a harmful cyanobacterium, Microcystis aeruginosa. Water Res. **54**, 188–198. (10.1016/j.watres.2014.01.063) 24568788

[53] Pimentel JSM, Giani A. 2014 Microcystin production and regulation under nutrient stress conditions in toxic Microcystis strains. Appl. Environ. Microbiol. **80**, 5836–5843. (10.1128/aem.01009-14) 25038094 PMC4178597

[54] Wang S, Ding P, Lu S, Wu P, Wei X, Huang R, Kai T. 2021 Cell density-dependent regulation of microcystin synthetase genes (mcy) expression and microcystin-LR production in Microcystis aeruginosa that mimics quorum sensing. Ecotoxicol. Environ. Saf. **220**, 112330. (10.1016/j.ecoenv.2021.112330) 34020285

[55] Wood SA, Puddick J, Hawes I, Steiner K, Dietrich DR, Hamilton DP. 2021 Variability in microcystin quotas during a Microcystis bloom in a eutrophic lake. PLoS One **16**, e0254967. (10.1371/journal.pone.0254967) 34288957 PMC8294494

[56] Giovannoni SJ, Cameron Thrash J, Temperton B. 2014 Implications of streamlining theory for microbial ecology. ISME J. **8**, 1553–1565. (10.1038/ismej.2014.60) 24739623 PMC4817614

[57] Sabath N, Ferrada E, Barve A, Wagner A. 2013 Growth temperature and genome size in bacteria are negatively correlated, suggesting genomic streamlining during thermal adaptation. Genome Biol. Evol. **5**, 966–977. (10.1093/gbe/evt050) 23563968 PMC3673621

[58] Sarnelle O. 1992 Contrasting effects of Daphnia on ratios of nitrogen to phosphorus in a eutrophic, hard‐water lake. Limnol. Oceanogr. **37**, 1527–1542. (10.4319/lo.1992.37.7.1527)

[59] Sommer U. 1989 The role of competition for resources in phytoplankton succession. In In plankton ecology: succession in plankton communities (ed. U Sommer), pp. 57–106. Berlin, Germany: Springer. (10.1007/978-3-642-74890-5_3)

[60] De Senerpont Domis LN, Mooij WM, Huisman J. 2007 Climate-induced shifts in an experimental phytoplankton community: a mechanistic approach. In Shallow lakes in a changing world (eds RD Gulati, E Lammens, N De Pauw, E Van Dock), pp. 403–413. Dordrecht, The Netherlands: Springer. (10.1007/978-1-4020-6399-2_36)

[61] Hamilton EW III, McNaughton SJ, Coleman JS. 2001 Molecular, physiological, and growth responses to sodium stress in C 4 grasses from a soil salinity gradient in the Serengeti ecosystem. Am. J. Bot. **88**, 1258–1265. (10.2307/3558337) 11454626

[62] Moura C, Lollo P, Morato P, Amaya-Farfan J. 2018 Dietary nutrients and bioactive substances modulate heat shock protein (HSP) expression: a review. Nutrients **10**, 683. (10.3390/nu10060683) 29843396 PMC6024325

[63] Gerloff-Elias A, Barua D, MÃ¶lich A, Spijkerman E. 2006 Temperature- and pH-dependent accumulation of heat-shock proteins in the acidophilic green alga Chlamydomonas acidophila. FEMS Microbiol. Ecol. **56**, 345–354. (10.1111/j.1574-6941.2006.00078.x) 16689867

[64] Schmidt KC, Jackrel SL, Smith DJ, Dick GJ, Denef VJ. 2020 Genotype and host microbiome alter competitive interactions between Microcystis aeruginosa and Chlorella sorokiniana. Harmful Algae **99**, 101939. (10.1016/j.hal.2020.101939) 33218432

[65] Asadulghani Suzuki Y, Nakamoto H. 2003 Light plays a key role in the modulation of heat shock response in the cyanobacterium Synechocystis sp PCC 6803. Biochem. Biophys. Res. Commun. **306**, 872–879. (10.1016/s0006-291x(03)01085-4) 12821123

[66] Jackrel S, Kuijpers M. 2024 Data from: Intraspecific divergence within Microcystis aeruginosa mediates the dynamics of freshwater harmful algal blooms under climate warming scenarios. Dryad Digital Repository. (10.5061/dryad.g79cnp5xt) PMC11793963 39904380

[67] Kuijpers MCM, Quigley C, Bray N, Ding W, White J, Jackrel S. 2025 Supplementary material from: Intraspecific divergence within Microcystis aeruginosa mediates the dynamics of freshwater harmful algal blooms under climate warming scenarios. Figshare. (10.6084/m9.figshare.c.7644191) PMC11793963 39904380

